# Usefulness of ethiodized oil and gelatin sponge particles for delaying the washout of indocyanine green from the liver in swine

**DOI:** 10.1007/s11604-022-01315-9

**Published:** 2022-07-16

**Authors:** Ryota Tanaka, Tetsuo Sonomura, Masaki Ueno, Masataka Koike, Ayano Makitani, Hirotatsu Sato, Kodai Fukuda, Nobuyuki Higashino, Akira Ikoma, Shin-ichi Murata, Hiroki Minamiguchi

**Affiliations:** 1 Department of Radiology, Wakayama Medical University, 811-1 Kimiidera, Wakayama-shi, Wakayama, 641-8509 Japan; 2 Second Department of Surgery, Wakayama Medical University, 811-1 Kimiidera, Wakayama-shi, Wakayama, 641-8509 Japan; 3 Department of Human Pathology, Wakayama Medical University, 811-1 Kimiidera, Wakayama-shi, Wakayama, 641-8509 Japan

**Keywords:** Indocyanine green, Ethiodized oil, Gelatin sponge particles, Infrared camera system, Intraoperative navigation

## Abstract

**Purpose:**

To assess the effect of ethiodized oil (EO) and gelatin sponge particles (GS) on delaying the washout of indocyanine green (ICG) from the liver in swine.

**Methods:**

Fifteen swine were divided into 3 groups: injection of a mixture of ICG and water-soluble contrast medium (CM) followed by embolization with GS (group A), injection of a mixture of ICG and EO (group B) and injection of a mixture of ICG and EO followed by embolization with GS (group C). The liver surface was observed using an infrared camera system during and at 1, 2, 3, and 6 h after the procedure to measure ICG contrast. Livers were removed at 6 h for histopathological examination.

**Results:**

The contrast ratio between injected and non-injected regions at 6 h was 1.45 ± 0.44 in group A, 1.89 ± 0.37 in group B, and 3.62 ± 0.76 in group C. The contrast ratio in group C was significantly greater than that in groups A and B (*P* = 0.032 and 0.033, respectively).

**Conclusions:**

EO and GS delayed the washout of ICG from the liver in swine and may extend intraoperative navigation in clinical use.

**Condensed abstract:**

Indocyanine green (ICG) mixed with ethiodized oil (EO) was injected into the left hepatic artery in swine, and the artery was embolized with gelatin sponge particles (GS). We confirmed that ICG remained in the liver parenchyma up to 6 h after the procedure. EO and GS delayed the washout of ICG from the liver in swine.

## Introduction

Anatomical liver resection is referred to as the complete removal of the tumor-bearing Couinaud’s segment, including ablation of the portal vein and parenchymal dissection along the hepatic vein that anatomically defines the boundary between segments [1]. It is considered a well-balanced mode of liver resection, especially for patients with hepatocellular carcinoma, as a potential curative approach [2, 3] and preserves the liver parenchyma [4]. For proper anatomical liver resection, the plane between segments should be identified, and the landmark hepatic veins and portal vein bifurcations should be identified intraoperatively using real-time ultrasonography [5, 6].

The results of laparoscopic hepatectomy are comparable to those of open surgery. Laparoscopic hepatectomy also reduced complications such as bleeding and surgical trauma, and decreased hospital stay [7, 8]. Additionally, laparoscopic hepatectomy provided better short-term outcomes, with a decreased incidence of postoperative ascites and liver failure, in patients with underlying liver disease and reduced liver function [9]. However, laparoscopic hepatectomy is more difficult than open surgery due to the use of two-dimensional images (no stereoscopic effect or depth of field) and a narrow visual field. Therefore, intraoperative liver segmentation is necessary for laparoscopic anatomical liver resection.

In conventional open liver surgery, the tattooing method [4, 10] and the glissonian approach [11] are commonly used to identify the hepatic segments, often in combination with indocyanine green (ICG). Recently, Ueno et al. reported the usefulness of hepatic artery embolization (HAE) with ICG to identify the liver segments for laparoscopic anatomical liver resection [12, 13]. They mixed ICG with a water-soluble contrast medium (CM) and needed to perform surgery on the same day, because ICG washes out within 24 h after injection. Tanaka et al. reported the usefulness of ethiodized oil (EO), a lipid-soluble contrast medium, for delaying the washout of ICG from the liver [14]. This enabled the surgeons to perform surgery the next day. Based on this experience, it is very important to understand the kinetics of ICG to facilitate liver resection. Therefore, the purpose of this study was to investigate the effect of EO and gelatin sponge particles (GS) on delaying the washout of ICG from the liver in swine.

## Materials and methods

The animal experiment was approved by the institutional ethics review committee and was performed in accordance with the ‘‘Act on the Protection and Management of Animals’’ and the ‘‘Standards for the Care and Storage of Laboratory Animals and Alleviation of Pain’’ of Japan.

The following materials were used for injection in this experiment: ICG (Diagnogreen® for injection, 2.5 mg/mL, Daiichi Sankyo, Tokyo, Japan), CM (Iopamiron® injection 370; Bayer Yakuhin, Osaka, Japan), EO (Lipiodol® 480 injection; Guerbet Japan, Tokyo, Japan), and an embolic material (GS; Gelpart® 1 mm 80 mg; Nippon Kayaku, Tokyo, Japan).

Fifteen healthy female swine weighing 50–52 kg were divided into 3 groups: injection of a 1:1 ICG-CM mixture followed by embolization with GS (group A), injection of a 1:1 ICG-EO mixture (group B) and injection of a ICG-EO mixture followed by embolization with GS (group C). The ICG-CM and ICG-EO mixtures were prepared using a pumping method with a three-way stopcock. The pumping frequency was 20 reciprocations in 20 s. GS was prepared by adding 10 mL of CM to 1 vial of Gelpart®.

Preanesthesia was conducted using 5 mg/kg ketamine and 0.08 mg/kg atropine sulfate, and general anesthesia was maintained with isoflurane gas via intubation. Cardiac and respiratory data were monitored throughout the procedures. A midline incision was made in the abdomen of the swine just below the xiphoid process of the sternum to expose the liver.

**Fig. 1:**
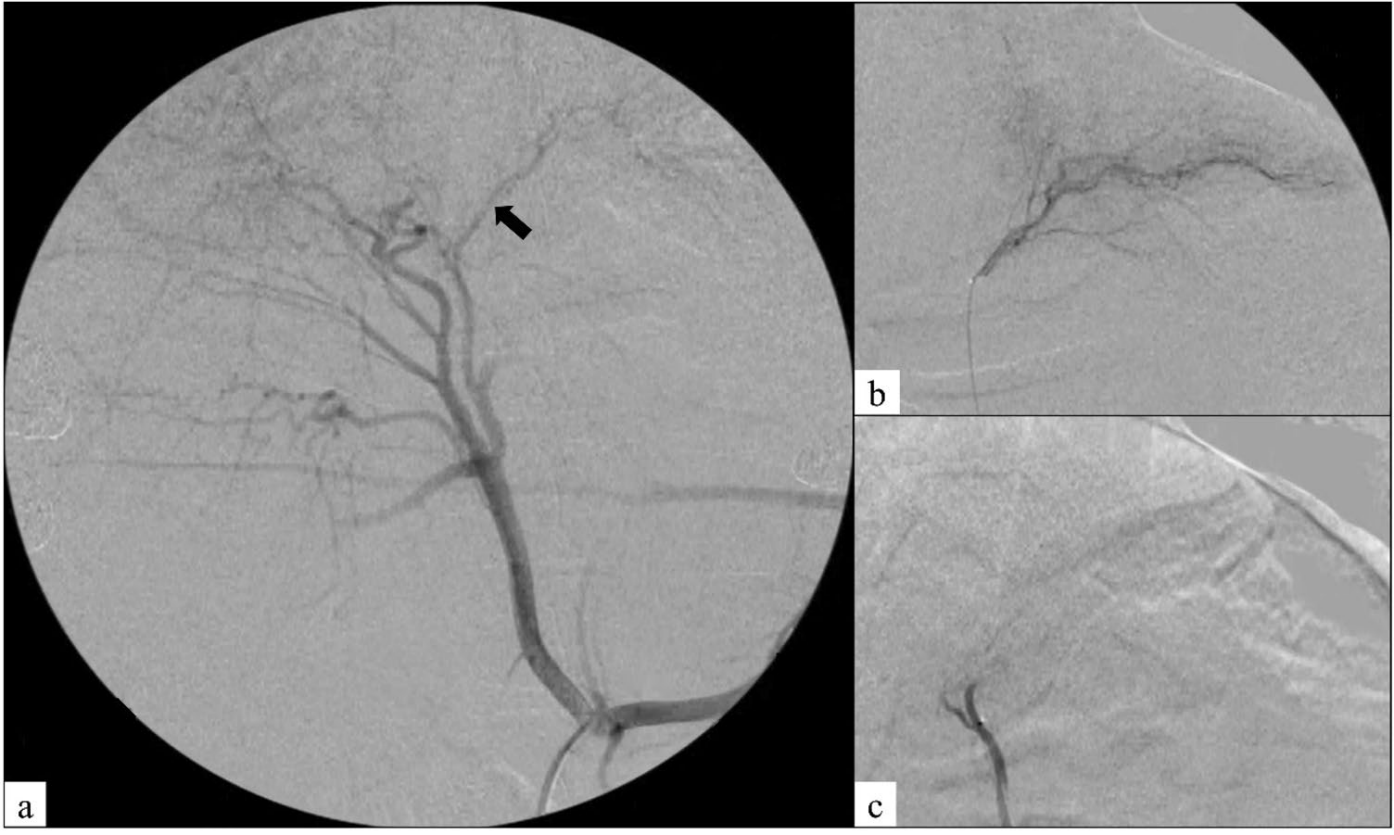
Angiography in a representative swine in group C. **a** Celiac arteriography showing a left hepatic artery (arrow) feeding a lateral left lobe. **b** Left hepatic arteriography before the procedure. **c** Left hepatic arteriography after the procedure showing complete occlusion of the left hepatic artery

Angiography was performed using an X-ray system (Allura Xper FD 20, Royal Philips Electronics, Amsterdam, Netherlands). First, a 6 Fr sheath (Radifocus Introducer II H, Terumo Clinical Supply, Gifu, Japan) was inserted via the right femoral artery, followed by a 4 Fr guide catheter (RC2, Medikit, Tokyo, Japan). Next, a 1.9 Fr microcatheter (Tellus, Asahi Intecc, Aichi, Japan) was inserted through the guide catheter, and the microcatheter was advanced into a left hepatic artery feeding a lateral or medial left lobe using a 0.016 in. microguidewire (Meister, Asahi Intecc, Aichi, Japan). Four mL of 1:1 ICG-CM mixture was injected into the left hepatic artery through a microcatheter in group A, and 4 mL of 1:1 ICG-EO mixture was injected in groups B and C. In a preliminary experiment, we confirmed that 4 mL of 1:1 ICG-EO mixture could be injected into a left hepatic artery without stagnation. Therefore, in all three groups, the injection volume of mixtures was set at 4 mL to ensure the amount of ICG was constant. Next, the left hepatic artery was embolized with GS in groups A and C (Fig. 1). The endpoint of embolization with GS was defined as complete occlusion of the hepatic artery.

**Fig. 2:**
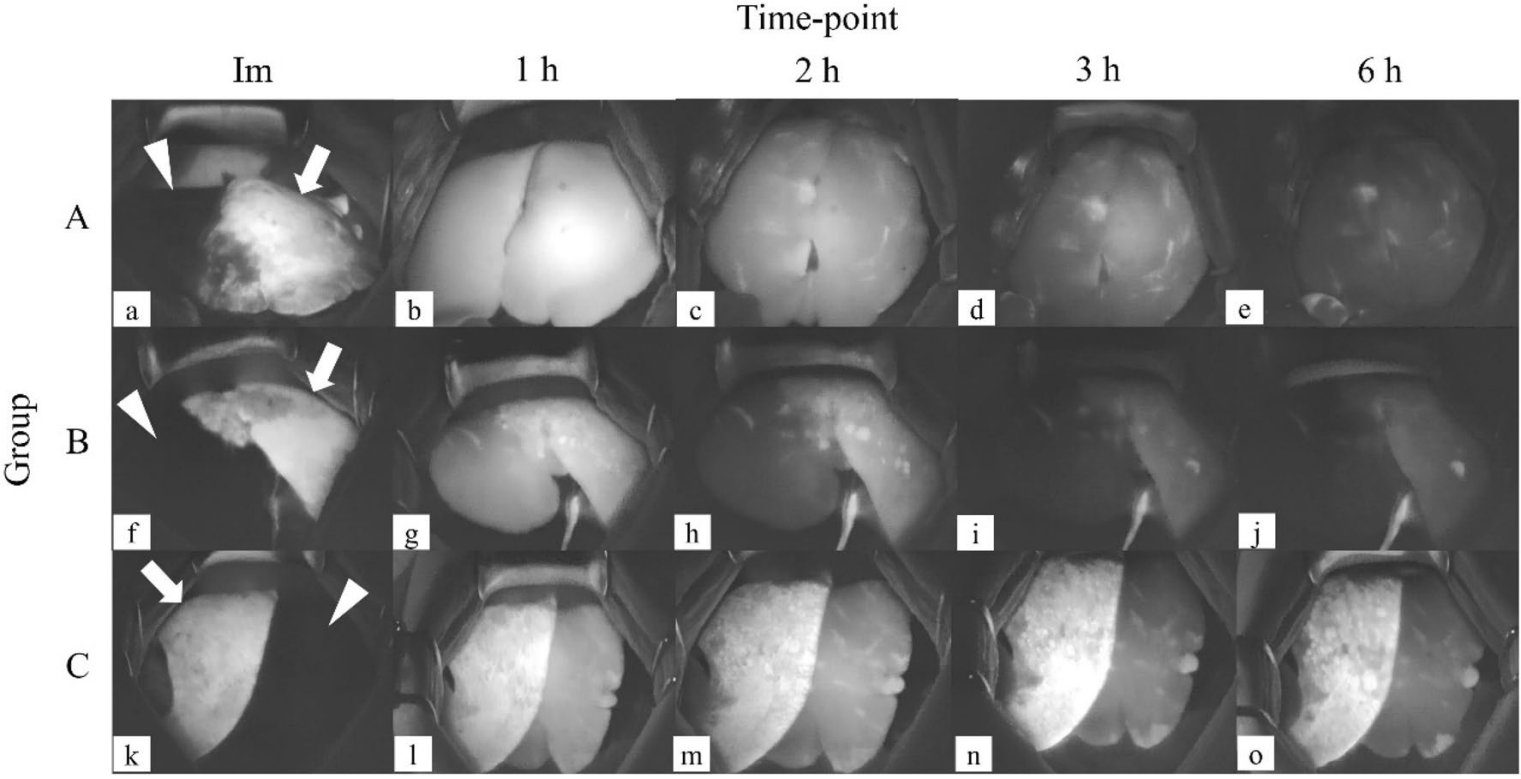
Representative images obtained using the infrared camera system showing the changes in the liver surface of the lateral or medial left lobe over time in one swine per group. The indocyanine green (ICG)-injected regions are shown in white (arrows) and non-injected regions in black (arrowheads). The regions with ICG are shown in a gradation of white to gray, depending on the amount of ICG. The retention of ICG is clearer in group C (k–o) than in groups A (a–e) and B (f–j). Images were taken immediately after the procedure (a, f, k; Im), and at 1 h (b, g, l; 1 h), 2 h (c, h, m; 2 h), 3 h (d, i, n; 3 h), and 6 h (e, j, o; 6 h) after the procedure

The liver surfaces of the lateral or medial left lobe were observed using an infrared camera system (pde-neo; IMI., Saitama, Japan) immediately after and at 1, 2, 3, and 6 h after the procedure to assess the intrahepatic retention of ICG (Fig. 2).

The livers were removed 6 h after the procedure and fixed with formalin. Hematoxylin/eosin staining was performed to assess the histopathological changes of the livers. Pathology specimens were cut using a knife and one sample per animal was assessed by a pathologist.

Images of the liver surface were analyzed using image-editing software (Photoshop CS6; Adobe Systems Inc., San Jose, CA, USA). The contrast value was quantified by grayscale analysis, and the contrast ratio was calculated by differentiating the region where the ICG mixture was injected from the region where it was not injected.

Statistical analyses were performed using JMP software (version 14.1). Comparisons among the three groups were made using the Steel–Dwass method. *P* < 0.05 was considered statistically significant. Values are presented as the mean ± standard deviation.

## Results

**Fig. 3:**
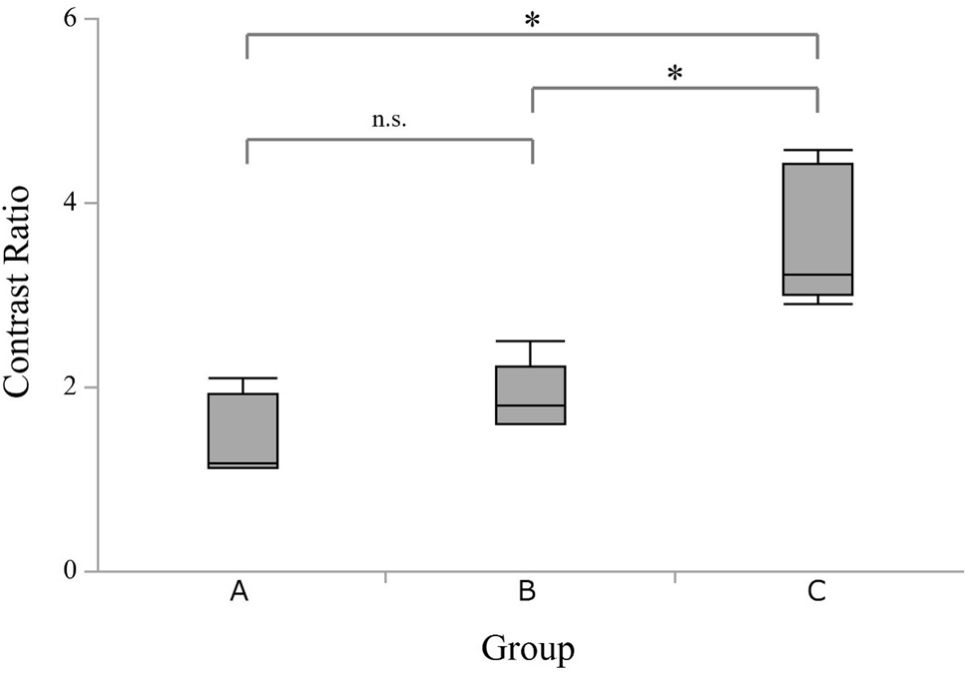
Quantitative analysis of the contrast ratios for indocyanine green at 6 h after the procedure in each group. The contrast ratio was significantly greater in group C than in groups A and B (*P* = 0.032 and 0.033, respectively), but was not significantly different between groups A and B (*P* = 0.42). *Significantly different at *P* < 0.05. n.s. = not significant

All the procedures were completed successfully in all 15 swine. However, the contrast values of ICG could not be evaluated at some time-points in 4 swine because of a problem with the recording device. The contrast values and contrast ratios of the ICG-injected and non-injected regions immediately after and at 1, 2, 3, and 6 h after the procedure are shown in Table 1. There was no significant difference in contrast ratios immediately after and at 1, 2, 3 h after the procedure. The contrast ratios (mean ± standard deviation) at 6 h after the procedure were 1.45 ± 0.44 in group A, 1.89 ± 0.37 in group B, and 3.62 ± 0.76 in group C (Table 2). The contrast ratio was significantly greater in group C than in groups A and B (*P* = 0.032 and 0.033, respectively). The contrast ratio was not significantly different between groups A and B (*P* = 0.42; Fig. 3).

**Table 1:** Contrast values and contrast ratios for the ICG-injected and non-injected regions in the individual swine in each group

Group	Swine	Contrast value (a)					Contrast value (b)					Contrast ratio				
		ICG-injected region					Non-injected region					a / b				
	No	Im	1 h	2 h	3 h	6 h	Im	1 h	2 h	3 h	6 h	Im	1 h	2 h	3 h	6 h
A	1	250	186	124	86	76	78	130	90	88	67	3.21	1.43	1.38	0.98	1.13
	2	B	B	B	152	95	B	B	B	71	45	B	B	B	2.14	2.11
	3	161	109	56	44	34	33	73	41	31	29	4.88	1.5	1.37	1.42	1.17
	4	159	195	122	82	51	31	133	114	74	5	5.13	1.47	1.07	1.11	1.13
	5	B	174	182	153	124	B	161	137	133	72	B	1.08	1.33	1.15	1.72
B	6	B	247	254	196	180	B	114	135	94	92	B	2.17	1.88	2.09	1.96
	7	80	167	102	77	54	30	85	82	43	34	2.67	1.96	1.24	1.79	1.61
	8	153	161	108	59	53	30	112	48	34	33	5.1	1.4	2.3	1.7	1.6
	9	120	179	194	171	102	25	160	123	113	57	4.8	1.12	1.58	1.51	1.79
	10	75	194	172	172	157	30	143	110	78	63	2.5	1.36	1.56	2.21	2.49
C	11	B	B	B	199	160	B	B	B	54	35	B	B	B	3.69	4.57
	12	198	130	117	124	120	38	48	33	32	28	5.21	2.71	3.55	3.86	4.29
	13	142	188	168	194	218	32	139	125	82	70	4.44	1.35	1.34	2.37	3.11
	14	160	185	175	194	190	31	119	89	58	59	5.16	1.55	1.97	3.34	3.22
	15	140	187	186	176	177	32	152	133	101	61	4.38	1.23	1.4	1.74	2.9

*Im* = Immediately after the procedure

*1, 2, 3 and 6 h* = time after the procedure

*B* = blank (the contrast value could not be determined owing to problems with the recording device)

*ICG* = indocyanine green

**Table 2:** Contrast values and contrast ratios for ICG at 6 h after the procedure in each group

Group		A	B	C
No. of swine		5	5	5
Contrast value				
ICG-injected region (a)	Mean ± SD	76 ± 35.55	109 ± 58.21	173 ± 36.43
	Median	76	102	177
Non-injected region (b)	Mean ± SD	52 ± 17.69	56 ± 24.28	51 ± 18.09
	Median	45	57	59
Contrast ratio (a / b)				
	Mean ± SD	1.45 ± 0.44	1.89 ± 0.37	3.62 ± 0.76
	Median	1.17	1.79	3.22

*ICG* = indocyanine green, *SD* = standard deviation

**Fig. 4:**
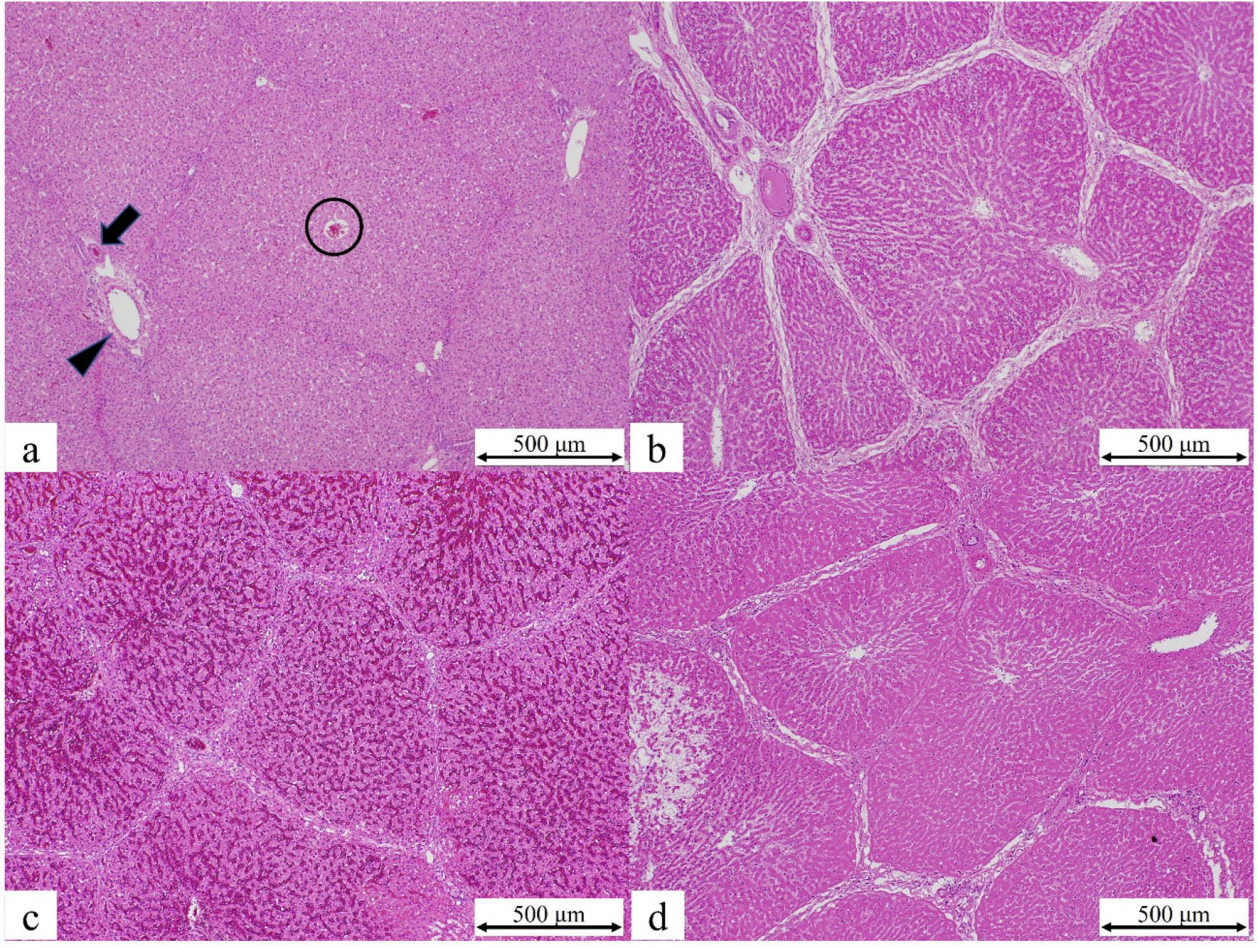
**a** Histopathological image of normal liver tissue showing the hepatic artery (arrow), the portal vein (arrowhead), and the central vein (circle) in group A. The lobular structures are intact. **b** Lobular neutrophil infiltration, hepatocyte disarray, periportal edema, and dilated sinusoids were visible in group B. **c** Dilatation and congestion of sinusoids were observed in group B. **d** Hepatocyte nuclear loss, which may indicate necrosis, was only observed in group C

Histologically, all three groups showed evidence of lobular neutrophil infiltration, hepatocyte disarray, periportal edema, and dilatation/congestion of the sinusoids; these features were more severe in group C than in groups A and B. Hepatocyte necrosis was observed in some regions of the liver in group C but not in groups A and B (Fig. 4).

## Discussion

In this study, we compared intrahepatic retention of ICG and pathologic features between group A (1:1 ICG-CM mixture and GS), group B (1:1 ICG-EO mixture), and group C (1:1 ICG-EO mixture and GS); a control group (1:1 ICG-CM mixture) was not considered, because it is well known that 1:1 ICG-CM mixture injected into the hepatic artery quickly washes out from the liver.

Immediately after the injection of ICG mixture, there was a clear difference in ICG retention between the injected and non-injected regions. However, by 1 h after injection, the difference between the two regions had attenuated (Fig. 2). This is possibly because some of the injected ICG skips first-pass metabolism in the liver to enter the systemic circulation, and hence returns to the liver parenchyma. The ICG present in the liver parenchyma washes out over time. Therefore, by 6 h after the procedure, there was little difference in contrast between the injected and non-injected regions in groups A and B. However, there was a clear difference in contrast between these regions in group C. In group C, ICG was trapped in the injection region, and the contrast ratio was significantly greater than that in groups A and B. This suggests that EO and GS delayed the washout of ICG from the liver.

There are two possible factors that contribute to the delayed washout of ICG. First, EO is a highly viscous, lipid-soluble, liquid embolic material that is often used in transcatheter arterial chemoembolization of hepatocellular carcinoma. When mixed with an aqueous solution, such as an anticancer drug, it forms an emulsion that flows into and stagnates in the portal vein branches and peribiliary vascular plexus around the tumor, thereby slowing the release of the anticancer drug [15, 16]. Similarly, EO emulsions containing ICG may stagnate in the blood vessels, inhibiting the uptake and excretion of ICG. In this study, we used a 1:1 ICG-EO mixture according to a previous case report [14]. However, Tanaka et al. reported that the percentage of water-in-oil emulsion in a 1:1 Epirubicin-EO mixture created using a pumping method with a three-way stopcock was about 17%, and about 70% in a 1:2 Epirubicin-EO mixture [17]. Because a water-in-oil emulsion is more viscous and has a greater embolization effect than an oil-in-water emulsion, the 1:2 ICG-EO mixture may be more appropriate for delaying the washout of ICG from the liver than the 1:1 ICG-EO mixture. Second, it is possible that embolization caused hepatocyte damage, which influenced the results. The pathological results revealed necrosis of hepatocytes in some areas in group C. Even in the absence of necrosis, some hepatocellular damage has occurred with a varying degree. ICG is an in vitro dye, and measuring the blood concentration of ICG over time after intravenous injection is used as a clinical test to evaluate the uptake of ICG from blood into the liver and the excretory capacity of the liver. In our experiment, embolization with GS was performed to prevent washout via blood flow after the injection of mixtures containing ICG. Considering the metabolic pathway of ICG and these pathological findings, it seems possible that the hepatocyte damage caused by embolization resulted in local hepatic dysfunction and inhibited the uptake and excretion of ICG, allowing ICG to be retained for a longer time.

Our study has three limitations to discuss. First, for animal welfare reasons, the number of animals was limited. Second, although the study revealed that EO and GS delayed the washout of ICG, the optimal concentration of ICG for human liver surgery remains unknown. Finally, the ICG contrast values were measured up to 6 h after the procedure, and it is unknown how long ICG remained in situ beyond this time. Due to the invasiveness of the procedure, it was difficult to observe the liver surface at 24 h after the procedure.

In conclusion, injection of ICG-EO mixture followed by embolization with GS effectively delayed the washout of ICG from the liver in swine and may help extend intraoperative navigation in clinical use.

## Abbreviations

ICG: Indocyanine green
HAE: Hepatic artery embolization
CM: Water-soluble contrast medium
EO: Ethiodized oil
GS: Gelatin sponge particles
